# Simulation of Upward Jump Control for One-Legged Robot Based on QP Optimization

**DOI:** 10.3390/s21051893

**Published:** 2021-03-08

**Authors:** Dingkui Tian, Junyao Gao, Chuzhao Liu, Xuanyang Shi

**Affiliations:** School of Mechatronical Engineering, Intelligent Robotics Institute, Beijing Institute of Technology, Beijing 100081, China; tiandingkui@bit.edu.cn (D.T.); 3120150091@bit.edu.cn (C.L.); shixuanyang@bit.edu.cn (X.S.)

**Keywords:** upward jumping, QP, ZMP, CoM, angular momentum, anti-slippage

## Abstract

An optimization framework for upward jumping motion based on quadratic programming (QP) is proposed in this paper, which can simultaneously consider constraints such as the zero moment point (ZMP), limitation of angular accelerations, and anti-slippage. Our approach comprises two parts: the trajectory generation and real-time control. In the trajectory generation for the launch phase, we discretize the continuous trajectories and assume that the accelerations between the two sampling intervals are constant and transcribe the problem into a nonlinear optimization problem. In the real-time control of the stance phase, the over-constrained control objectives such as the tracking of the center of moment (CoM), angle, and angular momentum, and constraints such as the anti-slippage, ZMP, and limitation of joint acceleration are unified within a framework based on QP optimization. Input angles of the actuated joints are thus obtained through a simple iteration. The simulation result reveals that a successful upward jump to a height of 16.4 cm was achieved, which confirms that the controller fully satisfies all constraints and achieves the control objectives.

## 1. Introduction

Jumping enables more flexibility and stronger terrain adaptability for robots in unstructured terrain. Therefore, jumping motion is an important athletic ability in humanoid technology.

To improve a robot’s jumping ability, Raibert and et al. designed a very innovative controller in the 1980s and realized the hopping motion of a hydraulic robot [1,2]. An existing legged robot can adjust the footing point by adjusting the step length and achieve jumping motion on flat ground [3]. Poulakakis and Grizzle developed a two-level hybrid controller that can be used on an spring-loaded inverted pendulum and induce a provably stable gait on an spring-loaded inverted pendulum [4]. Based on a point-foot robot with elastic legs and compliant hip joints, Hyon proposed a controller that does not require robot dynamics or any pre-planned trajectories, and used precise nonlinear dynamics to realize the robot’s continuous jump [5]. Haldane analyzed the ability of several arboreal mammals and robots, constructed a jumping robot using a leg mechanism that enhances the power modulation, achieved 78% of Gallago’s vertical jumping agility, and demonstrated the jumping ability of the constructed robot through experiments [6]. Yim achieved accurate and reliable leaping and landing on a narrow foot with the small one-legged jumping robot Salto-1P [7]. The above-mentioned robots have very light-weight legs, the torso of the robot accounts for the major proportion of the total mass and the torso mass of the robot is concentrated. Because these robots have point foot or negligible foot in size, these approaches cannot include constraints, such as stability, non-slippage, and limitation of angular acceleration in the launch or landing phase. Therefore, the robots in [1,2,3,4,5,6,7] cannot satisfy the requirements of humanoid robots’ jumping motion.

To investigate the jump motion of robots with the mass distribution of human legs, Nunez proposed a simplified mathematical model of a humanoid robot and applied a simple control scheme based on the sliding modes to achieve jumping motion [8]. Aoustin decomposed the jumping process into the launch phase, flight phase, and landing phase, derived the mathematical model for a one-legged robot without a foot [9] and with a massless foot [10], and applied torques to the actuated joints to keep the center of moment (CoM) of the mechanism always placed on the same vertical line. Geyer [11], Tamaddoni [12], and Xiong [13] simplified the jumping motion of the robot as a spring mass model and achieved the jumping motion of the robot. However, none of the above-mentioned approaches considered the stability and angular moment of the robot during the jump.

To theoretically solve the stability of the jumping robot, Barkan completed a jump simulation and experiment for a robot using the online trajectory generation method based on the Eulerian Zero Moment Point (ZMP) Resolution. The undesired torso angle fluctuation was greatly reduced without forcing the angular moment to be zero [14,15,16]. Kajita conducted a ZMP-based running pattern generation simulation at 3 km/h and an experiment on one-time hopping motion with both legs [17]. Barkan and Kajita prevented the robot from falling down by performing ZMP tracking for the desired trajectories instead of only constraining the ZMP inside the support polygon, but their algorithm has poor scalability and compatibility. This means that it is difficult to add various constraints, which should have been considered but were ignored in this scheme, such as the anti-slippage and limitation of the angle and angular accelerations, or add other tasks such as joint tracking. In [18,19,20], offline nonlinear optimization methods were used to generate the robot jumping trajectories and perform experiments on the robot, but it was difficult to use the controller on the robot in real time.

In jumping motion, stability is a prerequisite. The control of angular momentum, anti-slippage, and limitation of angular acceleration are also very important. Although existing studies have considered some of the above-mentioned problems, few studies have addressed all of them under a single framework. With consideration to the above-mentioned problems, a framework based on quadratic programming (QP) is proposed in this paper to achieve vertical jump motion for a robot. The main contributions of this study are as follows:

(1) A framework based on QP optimization for solving the vertical jump problem is proposed and successfully unifies the hard constraints and over-constrained goals in the jumping process.
(2) The restriction of ZMP instead of tracking, non-slippage, limitation of angular acceleration, are added to the optimization framework as hard constraints.

The simplified simulation model and main scheme of jumping are presented in Section 2. The preparation of the upward jump motion is introduced in Section 3. The real-time control of the jump and the results are disscussed in Section 4 and Section 5, respectively, followed by the discussion and conclusion.

## 2. Simplified Jump Model and Main Scheme of Jumping

### 2.1. Simplified Jump Model

**Figure 1:**
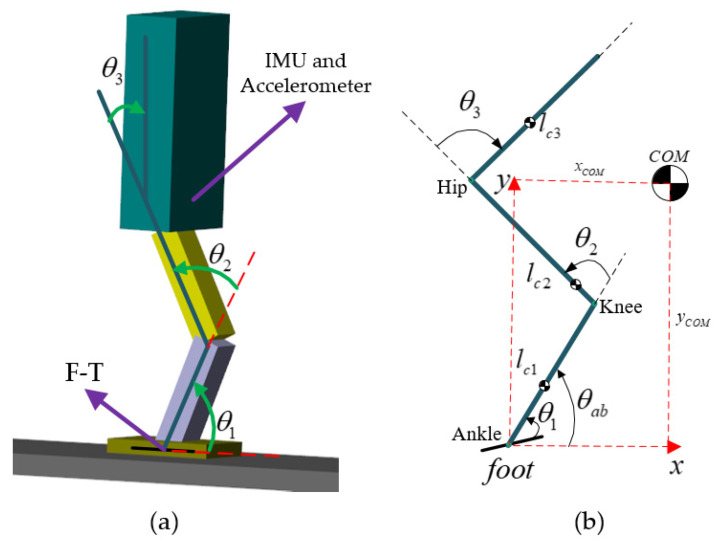
Humanoid robot model in simulation and its geometry. (**a**) Simulation model; (**b**) geometric model of the simplified robot.

In the design and manufacturing process of humanoid robots, the robots are generally arranged symmetrically in the sagittal plane. Hence, the first simplification for the robot is that the movement, external force, and torque on both sides of the robot are exactly the same. Although humanoid robots can possess more than 30 actuated joints, similar to humans, this study only considered the robot leg joints. Hence, the second simplification is that only the hip, knee, and ankle joints of the robot can move while the other joints are fixed. The two above-mentioned simplifications allowed us to establish the three-link robot presented in Figure 1 as our jump model.

As shown in Figure 1, in practical applications, the torso of the robot was equipped with an inertial measurement unit (IMU) and accelerometer used in the simulation to measure the absolute position and posture of the robot after the foot leaves the ground. In the ankle joint, six dimensional force torque (F-T) sensors are implemented to detect the forces and torques applied to the robot. The robot consists of three links (shrunk, thigh, and torso). In the stance phase, the robot possesses three actuated joints at the ankle, knee, and hip joint, which are denoted as $\theta_{1}^{}$, $\theta_{2}^{}$, and $\theta_{3}^{}$, respectively. The absolute pitch and positions of the frame attached to the ankle joint are denoted as $\theta_{ab}^{}$, $x_{f}$, and $y_{f}$, respectively. Although $\theta_{ab}^{}$ and $\theta_{1}^{}$ can be considered as identical in the stance phase, they are not necessarily equal in the flight phase. Additionally, $x_{com}$ and $y_{com}$ represent the CoM’s horizontal and vertical position in global coordinates.

Using the above notation, we define $\Theta =\left[\theta_{1},\theta_{2},\theta_{2}\right]$. By applying the Newton-Euler method, the dynamic equation of the robot in the stance phase can be obtained as follows:

(1) $$M\left(\Theta \right)\ddot{\Theta }+V(\Theta ,\dot{\Theta })+G\left(\Theta \right)=\tau$$

where $M\in R^{3\times 3}$ is the inertial matrix, $V\in R^{3}$ is the centripetal and Coriolis vector, $G\in R^{3}$ is the gravity vector, and $\tau \in R^{3}$ is the torque vector.

In the stance phase, it is assumed that the foot is always in contact with the ground and will not slide off, the robot possesses three degrees of freedom (DoF) and three actuated joints which are placed at respectively, the ankle, the knee and the hip joint, so it is fully actuated. During the flight phase, the robot possesses five DoFs and only two actuated joints, i.e., the knee and hip joint, which means that the robot is underactuated and is subjected to some restrictions, viz. two holonomic constraints resulting from the fact that CoM tracks a parabolic trajectory, and one non-holonomic constraint resulting from the angular momentum conservation.

The robot’s mass and inertia and the length of the ith link are denoted as $m_{i}^{}$, $I_{c}^{i}$, and $l_{i}$, respectively. The distance from CoM of each link of the robot to the proximal joint coordinate system is denoted as $l_{ci}$. The inertial parameters of the robot used in the simulation are listed in Table 1.

### 2.2. Main Scheme of Jumping

**Figure 2:**
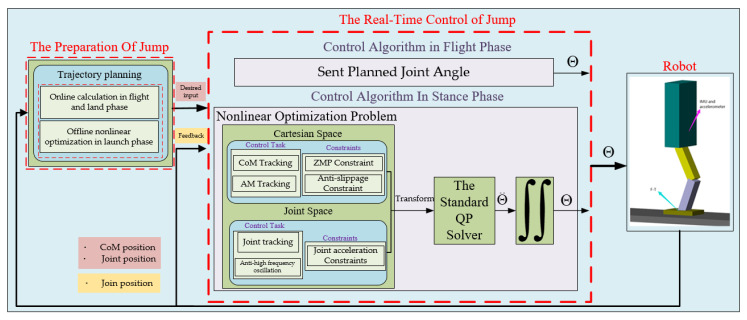
Control block diagram of robot; Θ and $\ddot{\Theta }$ are the actuated joint angle and acceleration vector, respectively.

The entire jumping process can be divided into three phases in chronological order, namely, the launch phase, flight phase, and landing phase [21]. The launch phase and landing phase can be merged into the stance phase because the foot is always in contact with the ground. As shown in Figure 2, the jumping motion of the robot consists of two parts: the first part is the preparation of the upward jump motion, which is divided into the offline trajectory generation in the launch phase and online trajectory generation in the flight phase and landing phase. The second part is the real-time control of jumping motion, which consists of flight phase control and stance phase control.

In the offline trajectory nonlinear optimization, the angular accelerations of the actuated joint are considered to be constant at each sampling interval (4 ms) [22,23]. Therefore, we selected the joints’ accelerations as the state vector. If the accelerations and torques of the joints’ reference trajectories are too large and exceed the capacity of the saturate torques of motors, it will be difficult to control the robot to track the reference trajectory. Therefore, we need to consider the indispensable constraints, such as the maximum accelerations and maximum torques. In order to minimize the integration of the joints’ accelerations and torques and avoid high-frequency oscillations, we penalized accelerations, torques and changes in torque respectively. So the launch problem can be described as a standard nonlinear optimization problem. By using a standard nonlinear optimization solver, the desired trajectories of the actuated joints can be obtained and the CoM can be calculated. To ensure the continuity of the actuated joint trajectory and the real-time calculation in the begining of the flight phase and landing phase, the trajectories are generated online and represented by cubic polynomial interpolation.

In the real-time control of the stance phase, the jumping motion of the robot can be simplified as the jumping of CoM, so the jumping goal needs to track the desired trajectory of CoM in *x*-axis direction and the *y*-axis direction. It is difficult to control the angular momentum of the robot respect to CoM to zero when the robot’s foot leaves the ground, the jumping goal needs to limit the angular momentum within a smaller range. To avoid the robot in undesirable configuration and high-frequency oscillations, the jumping goal must penalize the joints’ deviation from the desired trajectories and changes in joints’ accelerations, respectively. There are 4 control goals but only 3 control variables, i.e., the actuated joints’ accelerations, which leads us to unify this over-constrained jumping problem into a framework based on QP optimization with different weights and many constraints. We divided the control problem into Cartesian space and joint space. In Cartesian space, CoM tracking and angular momentum tracking are used as the task goal. Instead of implementing ZMP tracking for the robot, the ZMP is limited within the support polygon of the foot to prevent the robot from tipping over, which is used as a constraint. Additionally, the contact force is exerted within the friction cone to prevent slippage and is used as an additional constraint. In the joint space, joint tracking and the prevention of the joints’ high frequency oscillation are used as the task goals, while the joint accelerations within the limitation range are used as constraints. The task goals consist of nine equations and the robot has only three actuated joints, that is, three unknown variables, which is obviously an over-constrained and occasionally conflicting problem. To achieve a real-time solution in each sampling interval (4 ms), we were inspired by the solution method for the robot’s walking pattern in [24,25,26] and unified the indispensable constraints and over-constrained goals into a framework based on QP optimization with different weights in front of each objective to embody the priority of the task goals. Therefore, the actuated angular acceleration in each sampling interval can be estimated and the input angles of the actuated joints can then be obtained through a simple iterative process. In the real-time control of the flight phase, we only execute the planned angle of the actuated joints.

## 3. Preparation of Upward Jump Motion

The preparation of the upward jump motion comprises three parts: the launch phase, flight phase, and landing phase. The trajectory optimization in the launch phase is transcribed into a nonlinear optimization problem. We can specify the joints’ positions and easily solve the joints’ velocities on the basis of the conservation of angular momentum and linear momentum at the end of the flight phase. Joints’ positions and velocities at the end of the landing phase can be specified. Additionally, the positions and velocities of the joints can be obtained from the sampled joint data at the initial moments of the flight phase and the landing phase. We have obtained the position and velocity of joints at the initial and final moments in the flight and landing phase, and joints’ positions and velocities at intermediate time are unknown, so the trajectories in the flight and landing phase can be obtained using a polynomial instead of spline interpolation and Bessel interpolation. The first and quadratic polynomials are not sufficiently smooth, and the calculation of high-order polynomials is not easy and quick enough to implement on computer online. The cubic polynomial is smooth enough and quick to calculate online in real time, so the cubic polynomial was chosen.

### 3.1. Trajectory Planning in Launch Phase

We assumed that the acceleration of each joint between the two sampling intervals is approximately constant and selected the joint accelerations as the state vector. If the initial angle and velocity of the joint are known and the joint acceleration is solved, the angle and velocity of the actuated joints at each sampling time can be iteratively derived using the solved accelerations, initial given angle, and velocity; e.g., the angle and velocity at the (i + 1)th sampling time can be recursively obtained from the position, velocity, and solved acceleration at the ith sampling time, as follows:

(2) $$\left\{ \begin{array}{l} \theta_{i}^{\left[K+1\right]}=\theta_{i}^{\left[K\right]}+{\dot{\theta }}_{i}^{\left[K\right]}\Delta_{t}+{\ddot{\theta }}_{i}^{\left[K\right]}\frac{\Delta_{t}^{2}}{2}\\ {\dot{\theta }}_{i}^{\left[K+1\right]}={\dot{\theta }}_{i}^{\left[K\right]}+{\ddot{\theta }}_{i}^{\left[K\right]}\Delta_{t} \end{array}\right.$$

Here, $\theta_{i}^{\left[K\right]}$, ${\dot{\theta }}_{i}^{\left[K\right]}$, and ${\ddot{\theta }}_{i}^{\left[K\right]}$ denote the angle, velocity, and acceleration of the joint at the *K^th^* sampling time, and $\Delta_{t}$ is the sampling time equal to 4 ms in our calculations.

#### 3.1.1. Decision Vector

The decision vector is defined as follows:

(3) $$U={\left[{\ddot{\Theta }}^{\left[0\right]},{\ddot{\Theta }}^{\left[1\right]},\cdots ,{\ddot{\Theta }}^{\left[N-1\right]},{\ddot{\Theta }}^{\left[N\right]}\right]}^{T}$$

where ${\ddot{\Theta }}^{\left[K\right]}=\left[{\ddot{\theta }}_{1}^{\left[K\right]},{\ddot{\theta }}_{2}^{\left[K\right]},{\ddot{\theta }}_{3}^{\left[K\right]}\right]$, k indicates the $k^{th}$ discretized time interval, and N is the number of time intervals.

#### 3.1.2. Constraints in Launch Phase

(1) Initial constraints in launch phase

The initial restrictions in the joint space of the launch phase are characterized by the following relationships:

(4) $$\begin{array}{cc} \left\{ \begin{array}{l} \Theta =\Theta_{0}^{}\\ \dot{\Theta }={\dot{\Theta }}_{0}^{} \end{array}\right. & t=t_{initial}^{launch} \end{array}$$

where $\Theta_{0}^{}\in R^{3\times 1}$ and ${\dot{\Theta }}_{0}^{}\in R^{3\times 1}$ are the initial angle and velocity vector; $t_{initial}^{launch}$ is the initial time in the launch phase.

(2) Terminal constraint

Because this study focused on the vertical jump, the horizontal CoM component in the terminal launch phase was formulated as follows:

(5) $$\{\begin{array}{cc} \begin{array}{l} x_{com}=0\\ {\dot{x}}_{com}=0\\ {\ddot{x}}_{com}=0 \end{array} & t=t_{final}^{launch} \end{array}$$

where, $x_{com}$, ${\dot{x}}_{com}$, and ${\ddot{x}}_{com}$ denote the position, velocity, and acceleration of the horizontal CoM component; $t_{final}^{launch}$ is the terminal time of the launch phase.

After the foot of the robot leaves the ground, no external force acts on the robot except gravity and the CoM tracks a ballistic parabola trajectory, which means that the vertical force acting on the robot only needs to overcome gravity. Therefore, the sign of switching from the launch phase to the flight phase is the vertical acceleration component being equal to the acceleration caused by gravity. Because the vertical position and velocity of the CoM at the end of launch phase determines the shape of the ballistic parabola of the CoM in the flight phase, the terminal constraints in the vertical component are expressed as follows:

(6) $$\begin{array}{cc} \left\{ \begin{array}{l} y_{com}=Y_{final}^{launch}\\ {\dot{y}}_{com}=\sqrt{2gh}\\ {\ddot{y}}_{com}=-g \end{array}\right. & t=t_{final}^{launch} \end{array}$$

where $y_{com}$, ${\dot{y}}_{com}$, and ${\ddot{y}}_{com}$ denote the position velocity and acceleration of the vertical CoM component; g is the acceleration caused by gravity; h is the jump height of the CoM; $Y_{final}^{launch}$ is the vertical CoM component at the end of the launch phase.

If the foot leaves the ground, the robot system conserves the angular momentum relative to the CoM. Thus, successful landing becomes difficult when the angular momentum of the robot is very large, and a good course of action is to keep the angular momentum $L_{CoM}$ at zero when the robot foot leaves the ground, as follows:

(7) $$\begin{array}{cc} L_{CoM}=0 & t=t_{final}^{launch} \end{array}$$

(3) Constraints of horizontal CoM position

Because this study focused on upward jumping, the CoM position does not change in the horizontal direction, and the following relationships hold:

(8) $$\begin{array}{cc} \left\{ \begin{array}{l} x_{com}=0\\ {\dot{x}}_{com}=0\\ {\ddot{x}}_{com}=0 \end{array}\right. & t\in [0,t_{final}^{launch}) \end{array}$$

(4) Boundary constraints for joints

To better perform the practical jumping of the robot, the joint cannot exceed the angle, velocity, and acceleration limits, and the linear inequality constraints should be as follows:

(9) $$\begin{array}{cc} \left\{ \begin{array}{l} \Theta_{\min}^{}\leq \Theta \leq \Theta_{\max}^{}\\ {\dot{\Theta }}_{\min}^{}\leq \dot{\Theta }\leq {\dot{\Theta }}_{\max}^{}\\ {\ddot{\Theta }}_{\min}^{}\leq \ddot{\Theta }\leq {\ddot{\Theta }}_{\max}^{} \end{array}\right. & t\in [0,t_{final}^{launch}) \end{array}$$

where, $\Theta_{\min}^{}\in R^{3}$ and $\Theta_{\max}^{}\in R^{3}$ represent the lower and upper angle boundary, respectively; ${\dot{\Theta }}_{\min}^{}\in R^{3}$ and ${\dot{\Theta }}_{\max}^{}\in R^{3}$ represent the lower and upper joint velocity boundary, respectively; ${\ddot{\Theta }}_{\min}^{}\in R^{3}$ and ${\ddot{\Theta }}_{\max}^{}\in R^{3}$ denote the lower and upper joint acceleration boundary, respectively.

(5) Ground reaction force constraints

Throughout the launch phase, the vertical force $f_{Y}$ acting on the robot is always vertical to the ground, and the horizontal force $f_{X}$ is always opposite to the direction of motion and parallel to the ground, as follows:

(10) $$\begin{array}{cc} \left\{ \begin{array}{l} f_{X}=M_{t}{\ddot{x}}_{com}\\ f_{Y}=M_{t}\left({\ddot{y}}_{com}+g\right)\geq 0 \end{array}\right. & t\in [0,t_{final}^{launch}) \end{array}$$

where $M_{t}$ is the total mass of the robot. From Equation (8), we can get the horizontal force $f_{X}=0$ in the whole launch phase.

To ensure that the foot will not tip over and cause the robot to fall down, the ZMP should be kept inside the support polygon of the foot, which can be expressed as follows:

(11) $$l_{\min}\leq \begin{array}{cc} ZMP_{x}\leq l_{\max} & t\in [0,t_{final}^{launch}) \end{array}$$

To ensure that the foot does not slip and stays firmly on the ground, the horizontal force should not exceed the friction and cause the robot to slip, as follows:

(12) $$\begin{array}{cc} \left|f_{X}\right|\leq u_{s}f_{Y} & t\in [0,t_{final}^{launch}) \end{array}$$

where $u_{s}$ is the static friction coefficient. Because of $f_{X}=0$, Equation (12) can always be satisfied in the launch phase.

The maximum amplitude of the contact force must not exceed the maximum value $f_{\max}$ to avoid damaging the mechanical structure of the robot.

(13) $$\begin{array}{cc} \sqrt{f_{X}^{2}+f_{Y}^{2}}\leq f_{\max} & t\in [0,t_{final}^{launch}) \end{array}$$

(6) Torque constraints

With regard to the capacity of the motor and gearbox, it is meaningful to limit the output torques of the robot’s joints; therefore, the following torque constraints are imposed:

(14) $$\begin{array}{cc} \tau_{\min}\leq \tau \leq \tau_{\max} & t\in [0,t_{final}^{launch}) \end{array}$$

where $\tau_{\min}\in R^{3}$ and $\tau_{\max}\in R^{3}$ are the lower and upper boundary of the torque vector, respectively.

(7) Changes in vertical position

Similar to the launch process of a human, the robot should continuously expand its body to increase the CoM position throughout the launch phase. Therefore, the vertical position of the CoM in each sampling time is higher than the previous one, as follows:

(15) $$\begin{array}{cc} y_{com}^{\left[k\right]}\leq y_{com}^{\left[k+1\right]} & t\in [0,t_{final}^{stance}) \end{array}$$

where, $y_{com}^{\left[k\right]}$, and $y_{com}^{\left[k+1\right]}$ denote the vertical position of the CoM in the $k^{th}$ and ${\left(k+1\right)}^{th}$ discretized time interval.

#### 3.1.3. Cost Function

To minimize the total joint accelerations, the acceleration is penalized as follows:

(16) $$J_{dqq}=\sum_{k=1}^{N}\left({\left\|{\ddot{\Theta }}^{\left[k\right]}\right\|}^{2}\Delta_{t}\right)$$

The torques are penalized as follows:

(17) $$J_{\tau }=\sum_{k=1}^{N}\left({\left\|\tau^{\left[k\right]}\right\|}^{2}\Delta_{t}\right)$$

Torque changes are penalized to avoid high frequency oscillations, as follows:

(18) $$J_{\tau ch}=\sum_{k=1}^{N-1}{\left\|\tau^{\left[k\right]}-\tau^{\left[k+1\right]}\right\|}^{2}$$

#### 3.1.4. Nonlinear Optimization Problem

With consideration to the difference in priority, different weighting factors are added in front of each penalty. Therefore, the nonlinear optimization problem can be formulated as follows:

(19) $$\left\{ \begin{array}{l} \underset{U}{\min}\left(w_{ddq}J_{ddq}+w_{\tau ch}J_{\tau ch}+w_{\tau }J_{\tau }\right)\\ \begin{array}{cc} s.t. & equations\left(3\right) \end{array}\sim \left(14\right) \end{array}\right.$$

The trajectory planning parameters are summarized in Table 2 and Table 3.

**Figure 3:**
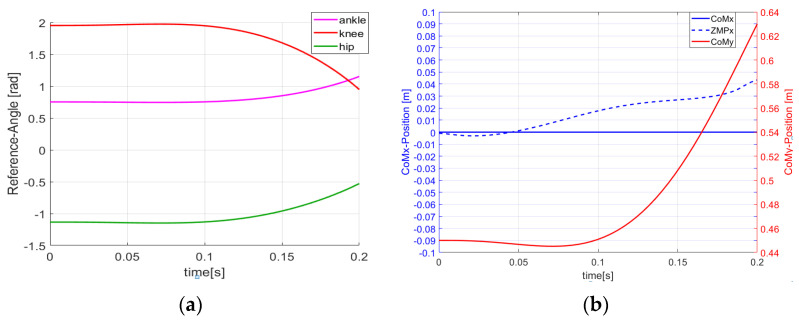
Trajectories of the joints and center of moment (CoM) in the launch phase. (**a**) trajectory of joints in the launch phase; (**b**) trajectories of CoM and zero moment point (ZMP) in the launch phase.

As shown in Figure 3, from the beginning to the end of the launch phase, the ankle, knee, and hip of the robot begin to accelerate and push the CoM to increase until the robot’s vertical component of acceleration is completely capable of overcoming gravity under various constraints, which is intuitively satisfactory for achieving human-like take-off motion. The ZMP is always inside the support polygon and the actuated joints always remain within the range of hardware capabilities.

### 3.2. Trajectory Planning in Flight Phase

When the robot’s foot leaves the ground in the launch phase, we can obtain the joint position $\Theta_{final}^{launch}$ and velocity ${\dot{\Theta }}_{final}^{launch}$ according to the joints’ code sensors, and calculate the position $\left(x_{com\to final}^{launch},y_{com\to final}^{launch}\right)$, velocity $\left({\dot{x}}_{com\to final}^{launch},{\dot{y}}_{com\to final}^{launch}\right)$, and angular momentum $L_{final}^{launch}$ with respect to the CoM.

When the robot touches the ground, we assume that the robot lands successfully and there is no slippage between the foot and the ground. Therefore, we can specify the angle vector $\Theta_{final}^{flight}$ with the torso perpendicular to the ground, and thereby the following relationship holds.

(20) $$\begin{array}{cc} \Theta =\Theta_{final}^{flight} & t= \end{array}t_{final}^{flight}$$

The robot’s angular momentum is conserved during the entire flight phase; therefore, the following equation holds:

(21) $$\begin{array}{cc} A_{1}\left(\Theta \right){\dot{\theta }}_{1}+A_{2}\left(\Theta \right){\dot{\theta }}_{2}+A_{3}\left(\Theta \right){\dot{\theta }}_{3}=L_{final}^{launch} & t=t_{final}^{flight} \end{array}$$

Because the torso is selected to be perpendicular to the ground, the velocity of the three actuated joints must satisfy the following equation:

(22) $$\begin{array}{cc} {\dot{\theta }}_{1}^{}+{\dot{\theta }}_{2}^{}+{\dot{\theta }}_{3}^{}=0 & t=t_{final}^{flight} \end{array}$$

During the entire flight phase, the horizontal component of the CoM is not affected by any external force; therefore, the horizontal speed of the CoM remains constant, as follows:

(23) $$\begin{array}{cc} {\dot{x}}_{com}\left(\Theta \right)={\dot{x}}_{com\to final}^{launch} & t=t_{final}^{flight} \end{array}$$

The angular velocity at the moment of landing can be easily solved according to Equations (20)–(23). To solve the actuated joint trajectories in real-time, $\theta_{2}$ and $\theta_{3}$ can be expressed by a cubic polynomial function.

**Figure 4:**
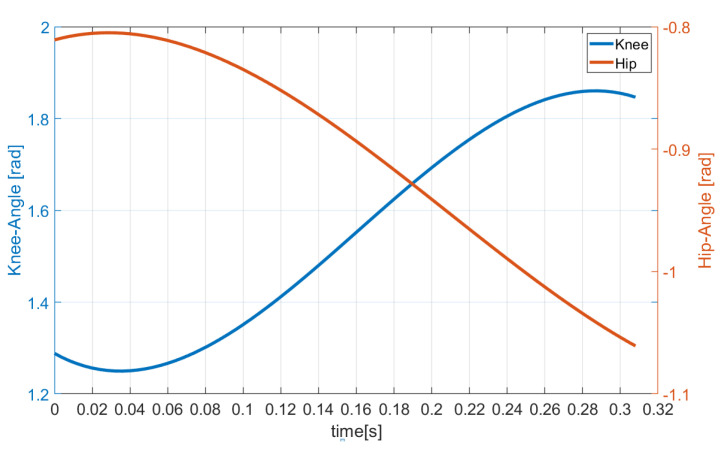
Trajectories of actuated joints in the flight phase.

As shown in Figure 4, during the initial phase of the flight, the two actuated joints continue to stretch owing to the large velocity caused by the launch phase. Then, to ensure that the robot is not in a singular configuration and prevent the impact force from causing great damage to the robot’s mechanical structure at touchdown, the actuated joints start to accelerate in the reverse direction and slowly move to the specified position.

### 3.3. Trajectory Planning in Landing Phase

When the robot’s foot contacts the ground again at the end of the flight phase, the foot is firmly placed on the ground. Therefore, we can obtain the joint position $\Theta_{initial}^{land}$ and velocity ${\dot{\Theta }}_{initial}^{land}$ through the joints’ codes, and calculate the position $\left(x_{com\to initial}^{land},y_{com\to initial}^{land}\right)$ and velocity $\left({\dot{x}}_{com\to initial}^{land},{\dot{y}}_{com\to initial}^{land}\right)$. The desired CoM trajectories and the hip angle can be expressed by using cubic polynomials, considering that the initial joint positions and velocities are known and the terminal positions and velocities of the CoM and hip are given.

**Figure 5:**
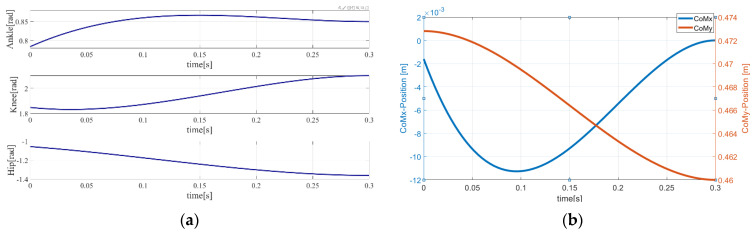
Trajectories of joints and CoM in landing phase. (**a**) Joint trajectories in the landing phase; (**b**) trajectory of CoM in the landing phase.

As shown in Figure 5, to avoid the rebound of the robot, which is caused by the touch-impact between the ground and the foot, and reduce the force acting on the foot, the actuated joints push the vertical CoM component to drop at the beginning of the landing phase. Then, the CoM starts to increase, which effectively prevents the robot from squatting too low and causing the knee to exceed the hardware capabilities.

## 4. Real-Time Control of Jump Motion

In the stance phase, we assume that the robot’s foot is always in contact with the ground without sliding. Hence, the three-link robot is fully actuated and the controllers in the launch phase and landing phase are the same. In the stance phase, the robot needs to track the well planned reference trajectories, limit angular momentum with respect to CoM into a small range, and satisfy many constraints, such as ZMP, anti-slippage and so on. In the iterative method for solving nonlinear equations, constraints cannot be considered. Therefore, the optimization method was chosen. The sampling interval for the jumping robot is 4ms or even shorter. The solutions cannot be obtained in real time by using nonlinear optimization method, the more efficient QP algorithm was selected. Eigen-QuadProg can solve the QP problem within 0.6 ms on the quadcore computer (Intel Celeron J1900, 1.99 GHz), which completely satisfy to obtain the solution online [27]. In the flight phase, the planned trajectories of two actuated joints are directly given to the servo control blocks at the actuator level.

### 4.1. Controller in Stance Phase

#### 4.1.1. Cartesian Space Controller

In the stance phase, we are concerned with whether the CoM of the robot can realize the kinematic performance of an inverted pendulum, as described in the previous section. Therefore, we applied PD feedback controllers such that the CoM trajectory could track the planned motion of the inverted pendulum in the vertical and horizontal direction, respectively.

The derivative and acceleration of the robot’s CoM in the horizontal directions is expressed as follows:

(24) $$\left\{ \begin{array}{l} {\dot{x}}_{com}=J_{x}\dot{\Theta }\\ {\ddot{x}}_{com}={\dot{J}}_{x}\dot{\Theta }+J_{x}\ddot{\Theta } \end{array}\right.$$

In the vertical directions, the CoM acceleration is expressed as follows:

(25) $$\left\{ \begin{array}{l} {\dot{y}}_{com}=J_{y}\dot{\Theta }\\ {\ddot{y}}_{com}={\dot{J}}_{y}\dot{\Theta }+J_{y}\ddot{\Theta } \end{array}\right.$$

where $J_{x}$ and $J_{y}$ are the Jacobian vectors of the CoM in the horizontal and vertical component, respectively.

In the horizontal direction, the input ${\ddot{x}}_{com}^{*}$ can be calculated as follows:

(26) $${\ddot{x}}_{com}^{*}=k_{px}\left(x_{com}^{d}-x_{com}\right)+k_{dx}\left({\dot{x}}_{com}^{d}-{\dot{x}}_{com}\right)+k_{ddx}{\ddot{x}}_{com}^{d}$$

The input ${\ddot{y}}_{com}^{*}$ is defined as follows:

(27) $${\ddot{y}}_{com}^{*}=k_{py}\left(y_{com}^{d}-y_{com}\right)+k_{dy}\left({\dot{y}}_{com}^{d}-{\dot{y}}_{com}\right)+k_{ddy}{\ddot{y}}_{com}^{d}$$

where $x_{com}^{d}$, ${\dot{x}}_{com}^{d}$, and ${\ddot{x}}_{com}^{d}$ are the desired position, velocity, and acceleration of the CoM in the horizontal direction; $y_{com}^{d}$, ${\dot{y}}_{com}^{d}$, and ${\ddot{y}}_{com}^{d}$ are the desired position, velocity, and acceleration of the CoM in the vertical direction.

The optimization problem for the desired horizontal trajectory of CoM tracking is expressed as follows:

(28) $$\underset{\ddot{\Theta }}{\min}{\left\|{\ddot{x}}_{com}^{}-{\ddot{x}}_{com}^{*}\right\|}^{2}$$

The vertical trajectory tracking can be formulated as follows:

(29) $$\underset{\ddot{\Theta }}{\min}{\left\|{\ddot{y}}_{com}^{}-{\ddot{y}}_{com}^{*}\right\|}^{2}$$

With regard to upward jumping, it is much easier for the angular momentum to remain close to zero before the foot leaves the ground, compared with compensating for the angular momentum by the motion of the actuated joints in the flight phase. Therefore, we need to add a controller to constrain the angular momentum close to zero. The angular momentum of the robot is given as follows:

(30) $$L_{CoM}=J_{L}\dot{\Theta }$$

The input ${\ddot{L}}_{CoM}^{*}$ can be calculated as follows:

(31) $${\dot{L}}_{CoM}^{*}=k_{pL}\left(L_{CoM}^{d}-L_{CoM}^{}\right)+k_{iL}\int_{0}^{t}\left(L_{CoM}^{d}-L_{CoM}^{}\right)dt$$

where $J_{L}$ denotes the Jacobian vectors of angular momentum, and $L_{CoM}^{d}$ is the desired angular momentum position. Because the ultimate goal is that the angular momentum should reach zero, we set $L_{CoM}^{d}$ equal to zero.

The optimization problem for the angular momentum in the launch phase can be expressed as follows:

(32) $$\underset{\ddot{\Theta }}{\min}{\left\|{\dot{L}}_{CoM}^{}-{\dot{L}}_{CoM}^{*}\right\|}^{2}$$

#### 4.1.2. Joint Space Controller

In addition to CoM tracking for the planned inverted pendulum’s kinematic trajectory, we also expect that the joints of the robot can track the planned joints’ trajectory.

The input ${\ddot{\Theta }}_{}^{*}$ can be calculated as follows:

(33) $${\ddot{\Theta }}_{}^{*}=k_{p\Theta }\left(\Theta_{d}^{}-\Theta \right)+k_{d\Theta }\left({\dot{\Theta }}_{d}^{}-\dot{\Theta }\right)+k_{dd\Theta }{\ddot{\Theta }}_{d}^{}$$

where $\Theta_{d}^{}\in R^{3}$, ${\dot{\Theta }}_{}^{d}\in R^{3}$, and ${\ddot{\Theta }}_{}^{d}\in R^{3}$ are the desired position, velocity, and acceleration vector of the actuated joints; $k_{p\Theta }\in R^{3\times 3}$, $k_{d\Theta }\in R^{3\times 3}$, and $k_{dd\Theta }\in R^{3\times 3}$ are gains.

The joint tracking problem can be formulated as follows:

(34) $$\underset{\ddot{\Theta }}{\min}{\left\|{\ddot{\Theta }}_{}^{}-{\ddot{\Theta }}_{}^{*}\right\|}^{2}$$

Penalizing the changes of the actuated joint accelerations is an effective way of preventing the high frequency oscillation of the actuated joints, as follows:

(35) $$\underset{\ddot{\Theta }}{\min}{\left\|{\ddot{\Theta }}_{}^{}-{\ddot{\Theta }}_{}^{N-1}\right\|}^{2}$$

where ${\ddot{\Theta }}_{}^{N-1}$ denotes the joint acceleration in the last sampling interval.

#### 4.1.3. Constraints

**Figure 6:**
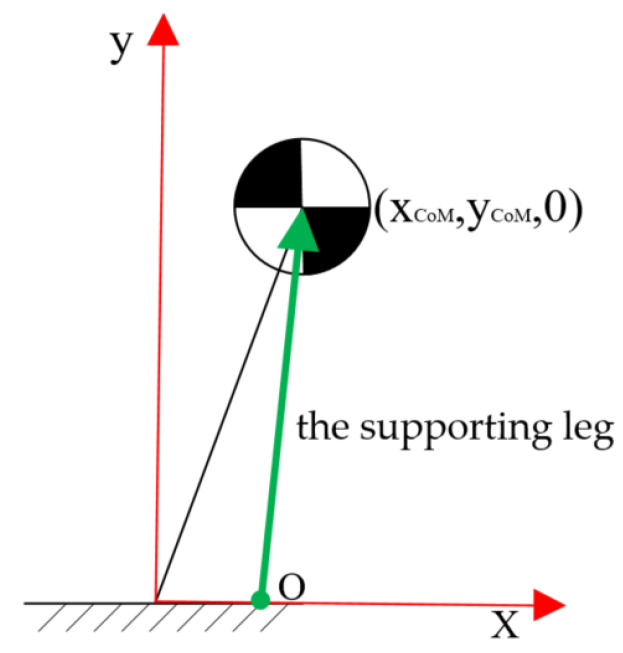
The inverted pendulum model.

As shown in the Figure 6, the supporting leg of the inverted pendulum intersects the *x*-axis at the support point O, whose coordinate is $\left(ZMP_{x},0,0\right)$, and the moment of CoM acting on the support point around the z axis is applied as follows:

(36) $$\tau_{z}=M_{t}\left({\ddot{y}}_{CoM}+g\right)\left(x_{CoM}-ZMP_{x}\right)-M_{t}{\ddot{x}}_{CoM}y_{CoM}$$

Since the moment of CoM acting on the support point around the z axis is zero, the equation of ZMP can be expressed as follows:

(37) $$ZMP_{x}=\frac{x_{com}g+x_{com}{\ddot{y}}_{com}-{\ddot{x}}_{com}y_{com}}{g+{\ddot{y}}_{com}}$$

To ensure the margin of control during the launch phase, a smaller support polygon constraints $l_{\min}$ and $l_{\max}$ are selected in the trajectory planning. Now, we select the support polygon constraints $L_{\min}$ and $L_{\min}$ according to the simulation model. Therefore, the ZMP constraints can be expressed as follows:

(38) $$L_{\min}\leq ZMP_{x}\leq L_{\max}$$

So ZMP are restricted as follows:

(39) $$L_{\min}\leq \frac{x_{com}g+x_{com}{\ddot{y}}_{com}-{\ddot{x}}_{com}y_{com}}{g+{\ddot{y}}_{com}}\leq L_{\max}$$

To prevent the horizontal slippage of the foot, the friction constraint is applied as follows:

(40) $$\left|{\ddot{x}}_{com}\right|\leq u_{s}\left({\ddot{y}}_{com}+g\right)$$

The joint acceleration cannot exceed the limitation; therefore, the upper and lower bounds of the state variables are restricted as follows:

(41) $${\ddot{\Theta }}_{\min}\leq \ddot{\Theta }\leq {\ddot{\Theta }}_{\max}$$

There are two task goals in Cartesian space and two task goals in joint space, but the robot has only three actuated joints, which is obviously an over-constrained and occasionally conflicting problem that can be solved through optimization with different weights applied to the optimization problems to distinguish the priority of different goals. Hence, the problem can be formulated as follows:

(42) $$\begin{array}{cc} \underset{\ddot{\Theta }}{\min} & \left[{\left\|w_{x}\left({\ddot{x}}_{com}^{}-{\ddot{x}}_{com}^{*}\right)\right\|}^{2}+{\left\|w_{y}\left({\ddot{y}}_{com}^{}-{\ddot{y}}_{com}^{*}\right)\right\|}^{2}+{\left\|w_{L}\left({\ddot{L}}_{CoM}^{}-{\ddot{L}}_{CoM}^{*}\right)\right\|}^{2}+{\left\|w_{\Theta }\left({\ddot{\Theta }}_{}^{}-{\ddot{\Theta }}_{}^{*}\right)\right\|}^{2}+{\left\|w_{fre\_osc}\left(\ddot{\Theta }-{\ddot{\Theta }}_{}^{N-1}\right)\right\|}^{2}\right]\\ s.t. & \left\{ \begin{array}{l} L_{\min}\leq \frac{x_{com}g+x_{com}{\ddot{y}}_{com}-{\ddot{x}}_{com}y_{com}}{g+{\ddot{y}}_{com}}\leq L_{\max}\\ \left|{\ddot{x}}_{com}\right|\leq u_{s}\left({\ddot{y}}_{com}+g\right)\\ {\ddot{\Theta }}_{\min}\leq \ddot{\Theta }\leq {\ddot{\Theta }}_{\max} \end{array}\right. \end{array}$$

where, $w_{x}$, $w_{y}$, $w_{\Theta }$, $w_{fre\_osc}$ and $w_{L}$ are weights for different optimization tasks.

#### 4.1.4. Transformation from Nonlinear Optimization Problem to QP Optimization Problem

The convergence of QP optimization is sufficiently fast for obtaining the solution in real time. Therefore, we selected QP as the method for solving this optimization problem. The problem formulated in Equation (42) can be transformed into a standard QP problem, and the following relationships can be obtained:

(43) $$\begin{array}{cc} \underset{\ddot{\Theta }}{\min} & \left[w_{x}^{2}{\left\|J_{x}\ddot{\Theta }+\left({\dot{J}}_{x}\dot{\Theta }-{\ddot{x}}_{com}^{*}\right)\right\|}^{2}+w_{y}^{2}{\left\|J_{y}\ddot{\Theta }+\left({\dot{J}}_{y}\dot{\Theta }-{\ddot{y}}_{com}^{*}\right)\right\|}^{2}+w_{L}^{2}{\left\|J_{L}\ddot{\Theta }+\left({\dot{J}}_{L}\dot{\Theta }-{\ddot{L}}_{CoM}^{*}\right)\right\|}^{2}+w_{\Theta }^{2}{\left\|\ddot{\Theta }-{\ddot{\Theta }}_{}^{*}\right\|}^{2}+w_{fre\_osc}^{2}{\left\|\ddot{\Theta }-{\ddot{\Theta }}_{}^{N-1}\right\|}^{2}\right]\\ s.t. & \left\{ \begin{array}{l} \left[\left(L_{\min}-x_{com}\right)J_{y}+y_{com}J_{x}\right]\ddot{\Theta }\leq -\left(L_{\min}-x_{com}\right)g-\left(L_{\min}-x_{com}\right){\dot{J}}_{y}\dot{\Theta }-y_{com}{\dot{J}}_{x}\dot{\Theta }\\ \left[-\left(L_{\max}-x_{com}\right)J_{y}-y_{com}J_{x}\right]\ddot{\Theta }\leq \left(L_{\max}-x_{com}\right)g+\left(L_{\min}-x_{com}\right){\dot{J}}_{y}\dot{\Theta }+y_{com}{\dot{J}}_{x}\dot{\Theta }\\ \left(J_{x}-u_{s}J_{y}\right)\ddot{\Theta }\leq -{\dot{J}}_{x}\dot{\Theta }+u_{s}{\dot{J}}_{y}\dot{\Theta }+u_{s}g\\ -\left(J_{x}+u_{s}J_{y}\right)\ddot{\Theta }\leq {\dot{J}}_{x}\dot{\Theta }+u_{s}{\dot{J}}_{y}\dot{\Theta }+u_{s}g\\ -\ddot{\Theta }\leq -{\ddot{\Theta }}_{\min}\\ \ddot{\Theta }\leq {\ddot{\Theta }}_{\max} \end{array}\right. \end{array}$$

### 4.2. Flight Phase

In the trajectory generation of the robot’s flight phase, it is considered that the robot is only subjected to gravity in the vertical direction, the linear momentum is in the horizontal direction, and the angular momentum with the CoM is conserved. Therefore, we only need to feed the well-planned angles to the actuated joint in each sampling period to achieve the desired ballistic dynamics of the robot and prepare for landing on the ground with the desired configuration.

## 5. Simulation Results

To validate the upward jump control method for the three-link planar biped robot, a computer simulation was conducted using Matlab/Simulink. In the simulation, $L_{\min}=-0.13$ and $L_{\max}=0.13$, which are determined by the size of the designed robot’s foot. The weights $w_{x}^{}$, $w_{y}^{}$ and $w_{L}^{}$ respectively determine the relative priorities of tracking of CoM in the directions of the *x*-axis and *y*-axis and tracking of angular momentum; a higher relative priority is indicated by a larger value. So we choose the larger $w_{x}^{}$ and $w_{y}^{}$. $w_{fre\_osc}^{}$ penalizes the change in the angular acceleration of the joints between two consecutive time steps, and $w_{\Theta }^{}$ determines the relative priority of the joints’ tracking of the desired trajectories. In the weighting matrices $w_{fre\_osc}^{}$ and $w_{\Theta }^{}$, only the diagonal elements corresponding to the joints are set to non-zero values. Firstly, because the priority of CoM is higher and the priority of angular momentum and joints is lower, $w_{x}=w_{y}=1$, $w_{L}^{}=0.0006$ and $w_{fre\_osc}^{}=w_{\Theta }^{}=\mathrm{diag}([0.0006,0.0006,0.0006])$ were chosen as the initial weights. Then, due to the poor tracking of CoM at the initial weights, $w_{x}$ and $w_{y}$ were manually increased according to the simulation results. The CoM tracked the desired trajectory very well at $w_{x}=w_{y}=1.6$. Because of the poor joints’ changes and large angular momentum, $w_{fre\_osc}^{}$, $w_{\Theta }^{}$ and $w_{L}^{}$ were manually increased under fixed $w_{x}$ and $w_{y}$, angular momentum fluctuated within a small range and the changes of joints’ positions are consistent with the desired trajectories at $w_{L}^{}=0.001$ and $w_{fre\_osc}^{}=w_{\Theta }^{}=\mathrm{diag}([0.001,0.001,0.001])$.

**Figure 7:**
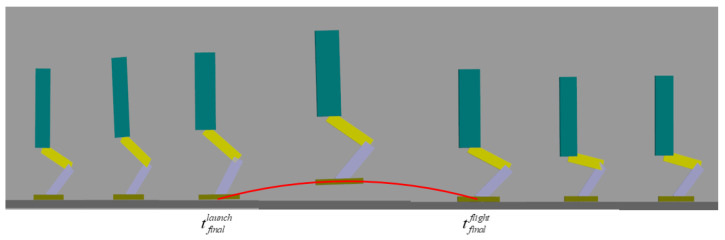
Screenshots of robot’s vertical jump motion.

**Figure 8:**
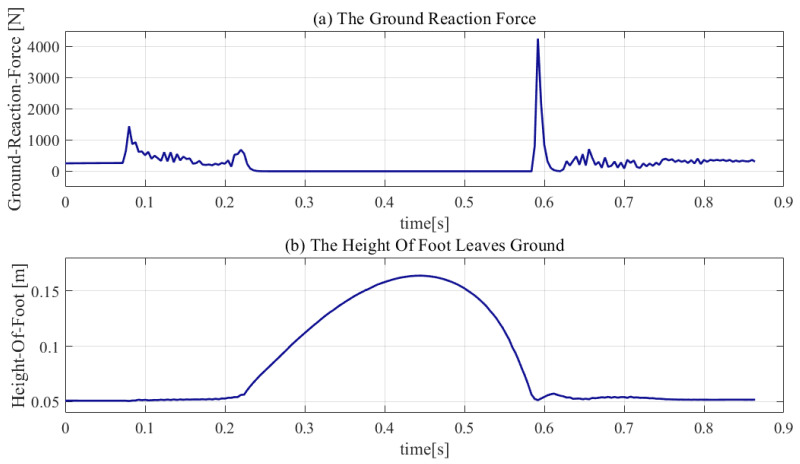
Reaction force and height of foot.

**Figure 9:**
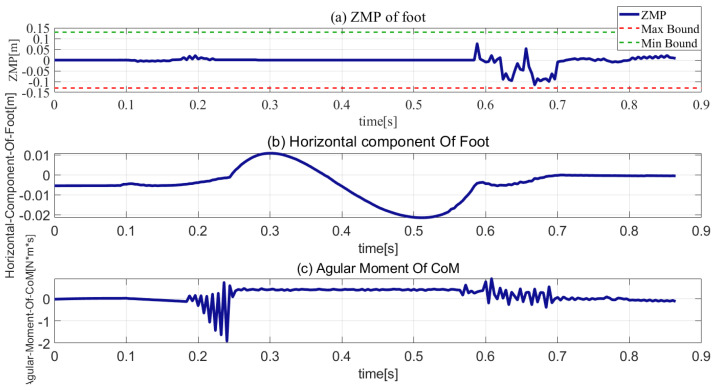
ZMP, horizontal foot position, and angular moment.

**Figure 10:**
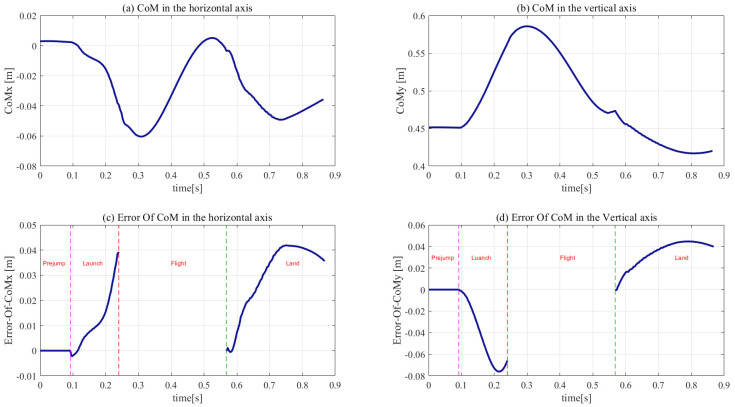
Trajectory of CoM and its simulation error.

**Figure 11:**
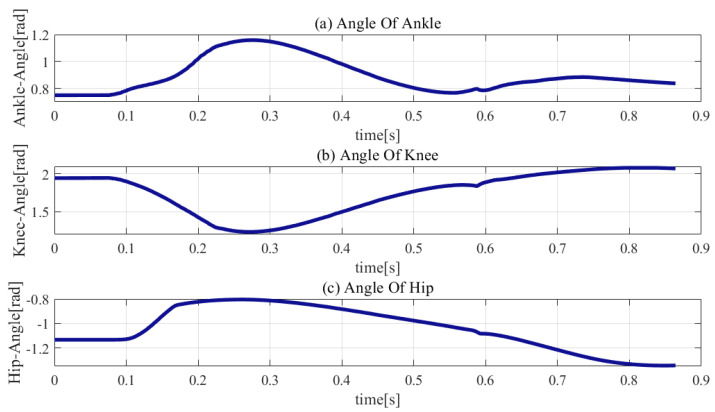
Joint trajectories in simulation.

The initial joint parameters, weights, and gains are listed in Table 4, Table 5 and Table 6, respectively. The simulation results are presented in Figure 7, Figure 8, Figure 9, Figure 10 and Figure 11.

Figure 7 and Figure 8 show that the robot performed a successful jump motion; the height of the upward jump was 16.4 cm and the time in the flight phase was 0.35 s. The zero ground reaction force in the flight phase indicates that the robot went through a successful launch phase. The successful landing of the robot demonstrates that the online planning of the flight phase trajectory is reasonable and effective. Moreover, the touch-down impact, which is harmful to the mechanical structure of the robot, can be observed. The touch-down impact was 4256 N, which is approximately 9.5 times the weight of the mechanical structure of robot’s body.

The response ZMP is illustrated in Figure 9a, the green, red, and blue plots are the upper and lower bounds of the support polygon and the ZMP plot, respectively. As can be seen, the ZMP response was always within the support polygon. Hence, it can be concluded that the robot was stable in the stance phase. Figure 9b shows the plot of the foot’s horizontal component. As can be seen, in the stance phase, the sliding of the robot’s foot was less than 0.5 cm. With regard to the touchdown impact, the movement of the foot in the horizontal component is almost negligible. Figure 9c shows that the angular momentum in the stance phase had a relatively small range, the maximum angular momentum did not exceed 2 N⋅m⋅s, and the angular momentum in the flight phase was less than 0.5 N⋅m⋅s. Therefore, it is concluded that fall and foot slippage prevention were successfully added to the constraints of the optimization problem, and the penalty function of the angular momentum was added to the cost function constraint’s angular momentum within a small range.

The trajectories in the horizontal and vertical position of the CoM and the corresponding errors are shown in Figure 10. The sharp edges in the CoM and the corresponding errors were observed at the moment of touch-down owing to the touch-down impact. Because the CoM was not controlled during the flight phase, the CoM error disappeared in the flight phase. The CoM’s error trajectories indicate that the maximum error in the horizontal position was 0.038 m at 0.244 s, while that in the vertical position was 0.076 m at 0.212 s. Therefore, it is concluded that the response trajectories are in good agreement with previously obtained results.

As shown in Figure 11, the joint trajectories exhibit smooth variation within the joints’ limitations, except at the initial landing phase owing to the touch-down impact. The hip, knee, and ankle have different degrees of sharp edges at the moment of touch-down. The sharp edges of the knee joint and ankle joint are larger, which demonstrates that the impact force causes more severe damage to the knee and ankle joints.

## 6. Discussion

Similar to the vertical jumping motion of humans, in the process of the robot’s vertical jump, constraints such as the stability, anti-slippage, and joint angular accelerations must be simultaneously considered. However, a method that considers all of these issues and integrates them into the jumping motion does not exist.

The proposed method is based on QP optimization and has good scalability. The above-mentioned restrictions, which must be considered, are unified into the proposed framework. In this paper, the trajectory optimization in the launch phase was first transcribed into an offline nonlinear optimization, and the trajectories in the flight phase and land phase were represented online using cubic polynomial interpolation. Moreover, an online QP-based framework was designed based on three-link dynamics to realize the robot’s upward jump motion, and successfully unified hard constraints such as the ZMP limitations and anti-slippage, and over-constrained tasks such as CoM, joint, and angular momentum tracking.

In this paper, an online QP optimization framework was designed based on three-link dynamics to realize a robot’s upward jump motion and successfully unify hard constraints, such as ZMP limitations and anti-slippage, and over-constrained tasks such as CoM, joint, and angular momentum tracking. The following results were obtained:

(1) The robot achieved a successful upward jump to the height of 16.4 cm.
(2) Throughout the stance phase, the ZMP was always limited inside the support polygon instead of tracking the desired value. Additionally, the movement of the foot in the horizontal component was almost negligible.
(3) The angular momentum in the stance phase had a relatively small range, the maximum angular momentum did not exceed 2 N·m·s, and the angular momentum in the flight phase was less than 0.5 N·m·s.

In future work, we will extend the jump algorithm to running robots. To this end, a substantial amount of work must be carried out. For example, the control of the simplified model in the jumping motion should be extended to the control of a full dynamics model in the running robot. In the support phase, the movement of the swinging leg should be controlled in addition to controlling the movement of the supporting leg. Owing to the compatibility and scalability of the QP-based framework, the extension of the jumping algorithm to running robots can be successfully incorporated into the proposed algorithmic framework.

## Figures and Tables

**Table 1:** Inertial robot parameters.

Link (*i*)	$m_{i}^{}$ (Kg)	$I_{c}^{i}$ (K·m^2^)	$l_{i}$ (m)	$l_{ci}$ (m)
Shank (1)	14.01	0.333	0.33	0.1454
Thigh (2)	13.04	0.718	0.34	0.1363
Torso (3)	16.38	0.8169	0.6	0.2141

**Table 2:** The scalar parameters in trajectory planning.

$t_{initial}^{launch}\left(s\right)$	$t_{final}^{launch}\left(s\right)$	$Y_{final}^{launch}\left(m\right)$	$g\left(kg/m^{2}\right)$	h(m)	$l_{\min}\left(m\right)$
0	0.2	0.63	−9.8	0.2	−0.1
$w_{ddq}$	$w_{\tau ch}$	$w_{\tau }$	$u_{s}$	$f_{\max}\left(N\right)$	$l_{\max}\left(m\right)$
1	0.001	0.01	0.6	1000	0.12

**Table 3:** The vector in the optimization of trajectory planning.

$\Theta_{0}^{}\left(rad\right)$.	${\ddot{\Theta }}_{0}^{}\left(rad/s\right)$	$\Theta_{\min}^{}\left(rad\right)$	${\dot{\Theta }}_{\min}^{}\left(rad/s\right)$	${\ddot{\Theta }}_{\min}^{}\left(rad/s^{2}\right)$
[0.75, 1.95, −1.13]	[0, 0, 0]	[0.43, 0, −1.56]	−[160, 360, 200]	−[360, 560, 500]
$\tau_{\min}\left(N\cdot m\right)$	$\tau_{\max}$	$\Theta_{\max}^{}\left(rad\right)$	${\dot{\Theta }}_{\max}^{}\left(rad/s\right)$	${\ddot{\Theta }}_{\max}^{}\left(rad/s^{2}\right)$
−[460, 600, 350]	[460, 600, 350]	[1.57, 2.28, 0]	[160, 360, 200]	[360, 560, 500]

**Table 4:** Initial joint parameters in simulation.

Link	Initial Position (rad)	Initial Velocity (rad/s)
Ankle	0.7494	0
Knee	1.9450	−0.0017
Hip	−1.1334	0

**Table 5:** Simulation weights.

$w_{x}^{}$	$w_{y}^{}$	$w_{\Theta }^{}$	$w_{fre\_osc}^{}$	$w_{L}^{}$
1.6	1.6	diag [0.001, 0.001, 0.0016]	diag[0.001, 0.001, 0.001]	0.001

**Table 6:** Simulation gains.

$k_{px}^{}$	$k_{dx}^{}$	$k_{ddx}^{}$	$k_{py}^{}$	$k_{dy}^{}$	$k_{ddy}^{}$
891	672	6	837.98	1726.27	5
$k_{pL}^{}$	$k_{iL}$	$k_{p\Theta }^{}$	$k_{d\Theta }^{}$	$k_{dd\Theta }^{}$	
91	16	diag ([26, 27, 25])	diag ([33, 36, 39])	diag ([6, 10, 5])	

## Data Availability

Data sharing not applicable.
